# Characterization of Probe Dynamic Behaviors in Critical Dimension Atomic Force Microscopy

**DOI:** 10.6028/jres.114.014

**Published:** 2009-08-01

**Authors:** Shaw C. Feng, Che Bong Joung, Theodore V. Vorburger

**Affiliations:** Manufacturing Engineering Laboratory, National Institute of Standards and Technology, Gaithersburg, MD 20899

**Keywords:** atomic force, cantilever, critical dimension, compliance, microscopy, nanometrology, probe modeling, scanned probe

## Abstract

This paper describes a detailed computational model of the interaction between an atomic force microscope probe tip and a sample surface. The model provides analyses of dynamic behaviors of the tip to estimate the probe deflections due to surface intermittent contact and the resulting dimensional biases and uncertainties. Probe tip and cantilever beam responses to intermittent contact between the probe tip and sample surface are computed using the finite element method. Intermittent contacts with a wall and a horizontal surface are computed and modeled, respectively. Using a 75 nm Critical Dimension (CD) tip as an example, the responses of the probe to interaction forces between the sample surface and the probe tip are shown in both time and frequency domains. In particular, interaction forces between the tip and both a vertical wall and a horizontal surface of a silicon sample are modeled using Lennard-Jones theory. The Snap-in and Snap-out of the probe tip in surface scanning are calculated and shown in the time domain. Based on the given tip-sample interaction force model, the calculation includes the compliance of the probe and dynamic forces generated by an excitation. Cantilever and probe tip deflections versus interaction forces in the time domain can be derived for both vertical contact with a plateau and horizontal contact with a side wall. Dynamic analysis using the finite element method and Lennard-Jones model provide a unique means to analyze the interaction of the probe and sample, including calculation of the deflection and the gap between the probe tip and the measured sample surface.

## 1. Introduction

Critical Dimension (CD) Atomic Force Microscopy (AFM) is currently a primary means to measure the geometric shapes of walls and trenches on the nanometer scale at device fabrication facilities in the semiconductor industry. As the widths of commercially available CD-AFM probes have become as small as 50 nm, the deformation of the probe tip during measurement may not be negligible. Although the prevailing method of CD-AFM tip width calibration includes such deformation in the “effective” tip width [1, 2], the compliance and deformation of the probes is expected to increase as the probe widths decrease. This raises the concern that probe tips become so compliant that the stability of the probe tips becomes a source of error in scanning a side wall [3]. To understand how the deformation might vary from one measurement configuration to another, it is necessary to develop a detailed computational model of probe-sample interaction and dynamic behaviors of the tip. The probe tip deflection relative to the beam is undetectable by the laser sensor and, therefore, could be a source of measurement error. There has been little analysis on the magnitude of the tip deflection and its geometric shape. Furthermore, the relative deflection of the tip and beam is different when the probe is scanning vertically and horizontally. In addition to probe deflection, the gap between the probe tip and the sample surface is another issue in contact probing. The relative position of an oscillating tip and a substrate as they approach each other to contact has not yet been analyzed. Lastly, there has been little analysis and estimation of the location of the measured point. Although all these factors are removed in the conventional method of CD-AFM tip calibration, we would like to understand their relative contributions and their potential for causing measurement instability. These phenomena also affect other types of AFM measurements of surfaces.

In related work, finite element models for static behavior and vibrational modes of top-down AFM have been reported by Song and Bhushan [4] and Feng, et al. [5]. Song and Bhushan’s models are for AFM on the micrometer scale. Feng’s model is for static analysis of the behaviors of a carbon nanotube-attached AFM on the nanometer scale. A Lennard-Jones model has been described and available from Sarid [6]. The material properties of a silicon probe are available from research results of Song and Bhushan [4]. Rayleigh damping analysis has been done, and research results are available [7]. On CD-AFM tip geometry, tip shape, round edges, and measurement uncertainty are available [1, 8]. While these results are useful in creating finite element models of a CD-AFM probe, they do not fully address dynamic behavior as discussed above.

In order to estimate the probe deflections due to surface intermittent contact and the resulting dimensional biases and uncertainties, we have developed finite element models for simulating the dynamic behavior of AFM cantilevers with a CD tip attached. Probe tip and cantilever beam responses to intermittent contact between the probe tip and sample surface are computed using the finite element method. Using the commercially available software system, Simulia,^2^ intermittent contacts with a wall and a horizontal surface are modeled and computed, respectively. Specifically, we characterized interaction forces as the tip is approaching a sample surface, using the Lenard-Jones theory. However, other forces, such as capillary and electrostatic forces are out of the scope of this paper. They can be analyzed separately and added later onto the Lennard-Jones forces. The finite element models are applied to model the probe, including the cantilever beam and probe tip, and to compute the beam deflection and probe tip deflection caused by the interaction force. The excitation frequency and amplitude at the beam base are also included in the model. With the calculated probe tip deflection and beam deflection during contacting, we analyze the relative deflection of tip and beam. We then compute the relative distance between the probe tip and the vertical or horizontal surface of the sample with the oscillating probe approaching and retracting. We thus can estimate the measured point on the surface based on the CD tip deflection and the gap. Finally, we consider the effect of a frictional force between a probe tip and a sidewall surface in contacting mode.

In this paper, there are several assumptions. We assume the following: the material deformation is elastic; the material properties, such as Young’s modulus, density, and damping ratio, are uniform in the whole probe; the tip end is a square, and the edge radius is uniform at the tip end; and dynamic friction is only considered for side-wall scanning.

## 2. Methods

**Fig. 1:**
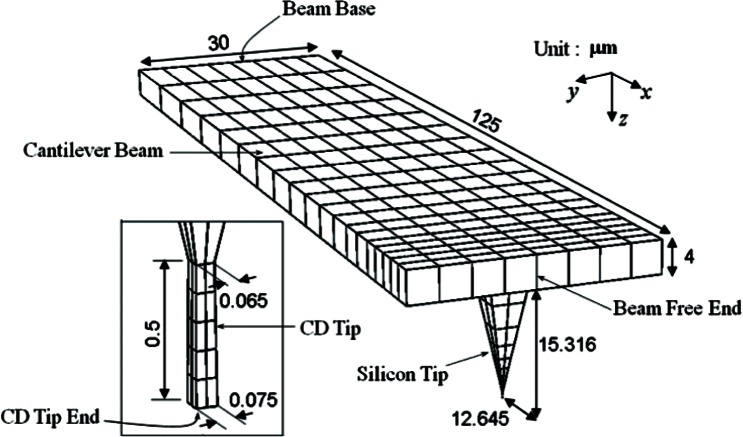
CD AFM probe model.

CD-AFM probes have specified shapes, dimensions, and material properties. They are operated with specified external excitations, and have reasonably well known boundary conditions at the beam base. Hence, a finite element model has been developed based on that information. Figure 1 shows a developed model. There are three components in the probe. A cantilever beam has one free end and a base. The base is where excitation is applied to the beam. The length of the beam is 125 μm. The width is 30 μm, and the thickness is 4 μm. The second component is a silicon tip. The exact dimensions are not critical. A CD tip is integrated into the end of the silicon tip. The CD tip has a 75 nm square end. The other end, which is attached to the silicon tip, is another square of 65 nm by 65 nm. The length of the CD tip is 0.5 μm. Such a geometry is used to touch a side wall of a feature in nanoelectronic manufacturing.

**Fig. 2:**
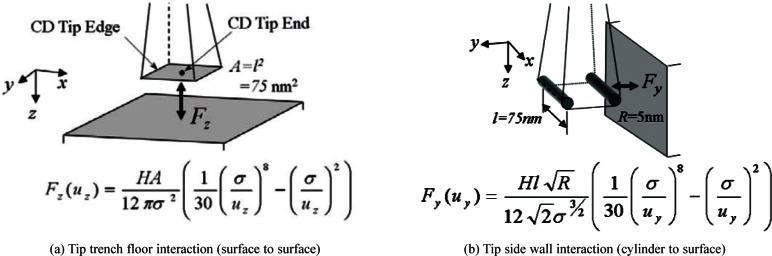
Force interaction model.

**Fig. 3:**
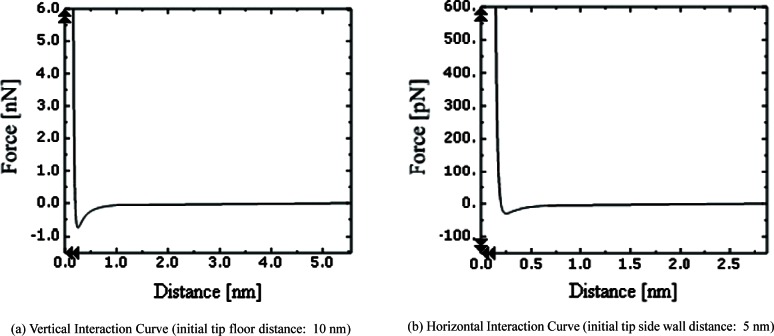
Tip-sample interaction forces in both Y and Z.

Regarding material properties, the Young’s modulus of silicon is 1.5 × 10^11^ Pa [4], the density is 2340 kg/m^3^ [4], the estimated damping ratio is 0.1, and the estimated Poisson ratio is 0.15. Rayleigh damping [7] is used in the model to approximate the damping in the probe. The interaction forces between the CD probe tip and sample surface are assumed to be governed by Lennard-Jones theory [6]. Figure 2(a) shows the geometry for a vertical interaction between the tip and the surface. The end of the tip, a 75 nm square, interacts with the horizontal surface of a sample. The Lennard-Jones equation is shown below the figure. *F_Z_* is the interaction force in the *Z* direction, and *u*_Z_ is the gap between the tip end and the horizontal surface. *F_Z_* is a Lennard-Jones force between two planes, one has a finite area of 75 nm by 75 nm and the other one is infinite [6]. We modeled the interaction as a concentrated force acting on the center of the CD tip end. It is also a point that is shared by the four finite elements at the tip end. H is the Hamaker constant of 4.25 × 10^(−19)^ J, and σ is the distance where the interaction potential energy is zero (0.35 nm) [9]. Figure 2(b) shows the geometry for a horizontal interaction where one of the cylindrical edges of the tip is interacting with the side wall. The equation is below the figure. *R* is the edge radius (5 nm) [1, 8], and *l* is the length of the edge (75 nm). *F_y_* is a Lennard-Jones force between the cylindrical edge and the plane. The cylinder has a finite length of 75 nm and the plane is infinite [6]. We modeled the force as a concentrated force acting on the center of the cylindrical edge. It is also a point that is shared by the two finite elements along the tip edge (see Fig. 1). Figure 3(a) shows tip-sample interaction forces in the *Z* direction due to the Lennard-Jones interaction, corresponding to the geometry of Fig. 2(a). The tip in its neutral position and the sample surface are initially set 12 nm apart. As the tip moves close to the sample surface, the tip is first attracted to the surface and then repelled. Likewise, Fig. 3(b) shows the Lennard-Jones forces for the horizontal interaction of Fig. 2(b).

The beam has a boundary condition at the beam base. The beam base is excited vertically at 320 kHz in the *Z* direction and oscillates horizontally at 5 kHz in the *Y* direction. 320 kHz is just below the natural frequency in the first mode of the probe. For both the vertical and horizontal orientations, the Lennard-Jones Force is attractive until the tip approaches within about 0.2 nm of the surface, then it becomes repulsive. For the horizontal interaction associated with probing sidewalls, the lateral oscillation of the beam enables the probe tip to snap out of the surface in case it snaps on to the surface.

**Fig. 4:**
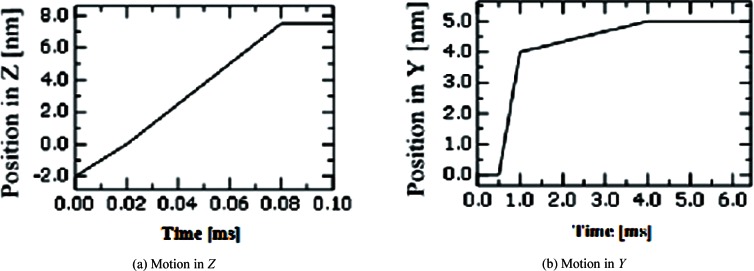
a) Probe-sample relative movement scenario in *Z* (a) for the vertical interaction case of Fig. 2a; b) probe-sample relative movement scenario in *Y* for the horizontal interaction case of Fig. 2b. These motions stop at 0.08 ms and 4 ms, respectively, after the tip and sample come into contact.

Figure 4 Sample-tip relative movement scenario in *Z* (a) for the vertical interaction case of Figure 2(a); sample-tip relative movement scenario in *Y* for the horizontal interaction case of Figure 2(b). These motions stop at 0.08 ms and 4 ms, respectively, after the probe and sample have come into contact.

For our analyses, we consider a case where the probe oscillates in the *Z* (italics for all variables unless they are abbreviations) direction and also moves monotonically toward the surface, either along the *Z* or *Y* direction, until the probe tip and sample surface make contact with each other. The relative movement between the probe tip and the sample is a function of time. Figure 4(a) shows the tip approaching a sample surface in the *Z* direction, and Figure 4(b) shows the tip approaching a side wall in the *Y* direction, respectively.

## 3. Finite Element Analysis and Results

### Finite element model

Finite element analysis has been applied down to nanometer scales with reasonably good results [10]. The meshed finite elements of the cantilever beam and tip are all hexahedrons. (C3D20, type name in SIMULIA [11]). There are 212 elements. Each element had been defined by 20 nodes having three degrees of freedom per node. Nonlinear interpolation is used between nodes to better approximate the bending effects than the assumption of linear interpolation. In the finite element analysis, the interaction force between the probe tip and the sample surface is modeled by a Connector [11] that has the force function of a Lennard-Jones curve according to the scanning orientation, e.g., the curve in Figs. 3(a) and 3(b).

### Modal analysis

The probe has an infinite number of modes. Table 1 lists the frequencies of the first five eigenmodes calculated with the model. It is necessary to know these natural frequencies. For scanning sample surfaces, the probe is vertically excited at a sub-resonant frequency (a frequency that is slightly below the first natural frequency). Exciting the probe below a natural frequency will result in high sensitivity on contacting the sample surface. Both amplitude and vibrational frequency will change when the CD tip end begins to contact a sample surface as explained below.

### Static behaviors of the CD-AFM probe

**Fig. 5:**
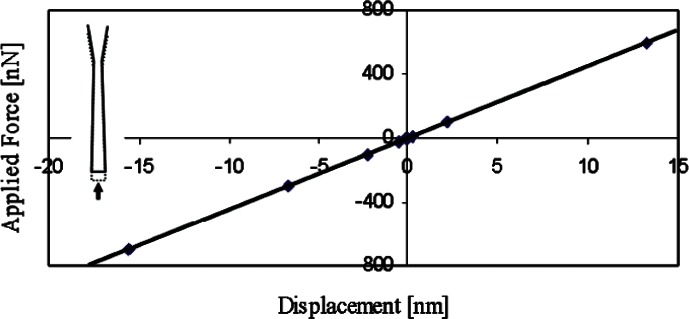
Force vs. tip displacement in *Z* at the CD tip end.

Both the cantilever beam and the CD tip deflect under bending moments. The bending moments are caused by contacting forces. Figure 5 shows the calculated flexural (vertical) spring constant (*K_Z_*) of the CD tip end, *K_Z_* is 45.07 nN/nm. A range of forces is vertically applied to the CD tip end, and deflections at the beam free end are calculated using the finite element method. Since the beam deflects linearly vs. force, the spring constant is the slope of the plotted line. Similarly, the lateral spring constant (*K_y_*) of the CD tip when lateral forces are applied is computed to 4.05 nN/nm. Notice that the lateral spring constant is more than 10 times smaller than the vertical spring constant, i.e., the probe is more than 10 times softer laterally than vertically. Furthermore, the cantilever beam can be twisted, i.e., the beam free end can rotate about the *X* axis when a lateral force is exerted during probing a side wall. The torsional spring constant relating the lateral force and the angular displacement about the *X* axis is 2.10 × 10^7^ nN/radian.

### Dynamic behaviors in probe tip and sample surface contacting

**Fig. 6:**
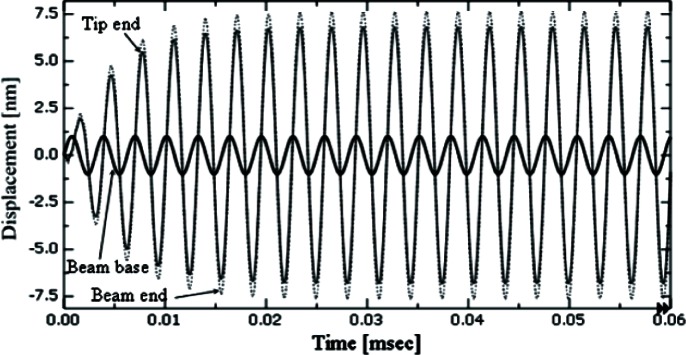
Free oscillations of the beam at 320 kHz. Oscillations at the tip end are slightly smaller than oscillations at the beam end because its distance from the beam base is smaller (see Fig. 1).

**Fig. 7:**
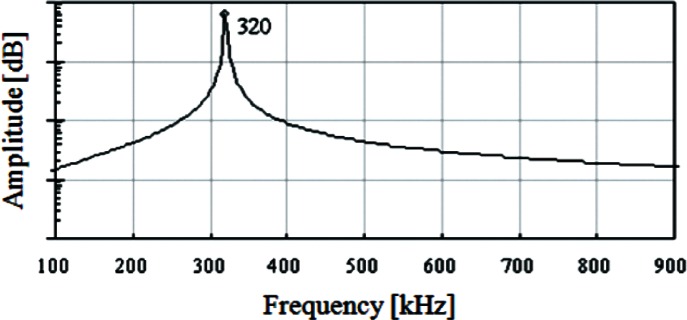
Frequency Spectrum of the steady state beam oscillation (Fig. 6).

**Fig. 8:**
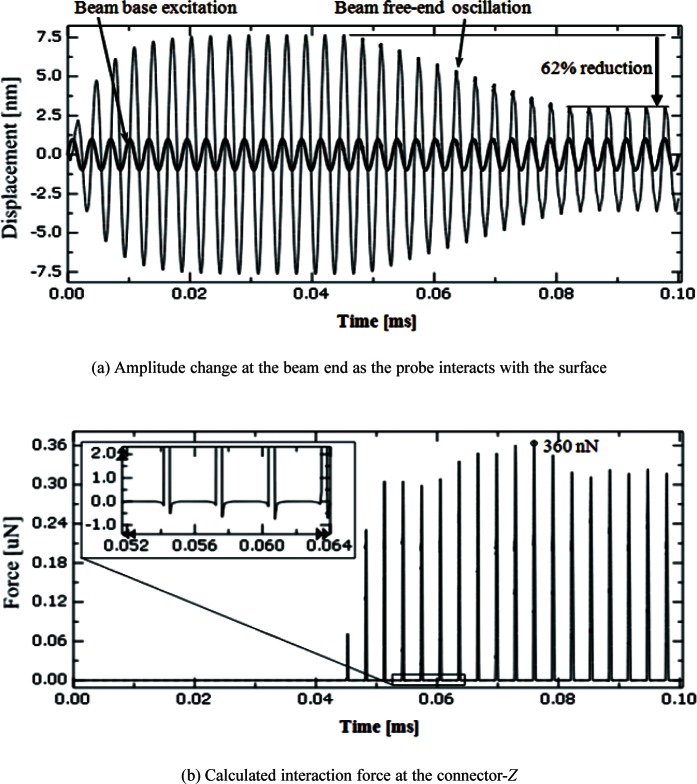
Amplitude change and interaction forces in .*Z*.

**Fig. 9:**
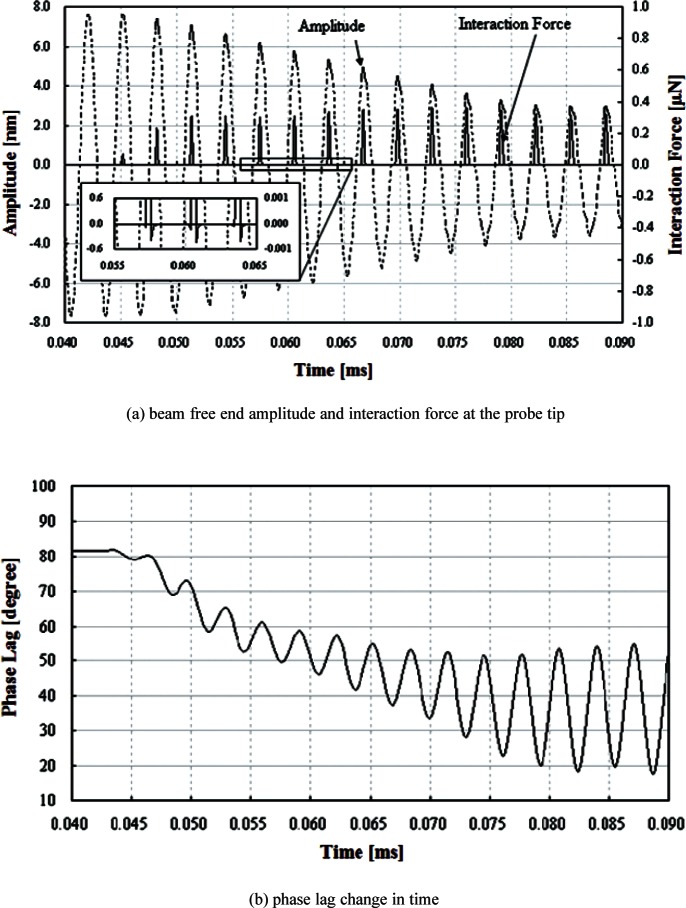
Amplitude and phase lag changes in the time of the contact in *Z* scanning.

The free oscillation of the probe is analyzed when it is excited at 320 kHz, a sub-resonant frequency. The analysis includes both amplitude and frequency of the beam free end. Figure 6 shows free oscillations in the time domain of the beam base (320 kHz with amplitude of 1 nm), the beam free end, and the CD tip end. Figure 7 shows the frequency spectrum of the driven steady state oscillation at the beam free end in the *Z* direction. The free oscillation takes place in the noncontact mode. When the probe and sample surface begin to interact, the amplitude of the beam oscillation begins to decrease. Figure 8 shows that the interaction force and amplitude change. Figure 8(a) shows that the CD tip and sample surface are close enough to experience an interaction force at a time of 0.04 ms. In the steady state, the amplitude at the beam free end decreases about 62 %, due to the interaction force at the CD tip. The force is primarily repulsive and in the *Z* (vertical) direction. Figure 8(b) shows that the maximum repulsive force exerted on the CD tip end is about 360 nN. In the inset, it shows that relatively small attractive forces are also exerted on the CD tip because the interaction includes attraction. Figure 9 shows relationships among interaction forces, amplitudes, and phase lag changes. Figure 9(a) shows the amplitude changes when the probe tip is making intermittent contacts with a horizontal sample surface. The amplitude decreases from about 7.6 nm to about 2.9 nm due to forces exerted on the tip in the *Z* direction. The contact force increases from null to about 360 nN. Note that repulsive forces are exerted only in a short period within a half of a full cycle when the tip and sample surface are in contact with each other. There is no force exerted in the other half of the full cycle. Since repulsive forces only exist in a half cycle, the amplitude is distorted from the sinusoidal shape. Also, note that there is a small attractive force when the tip snaps out of the surface as it is leaving. Figure 9(b) shows changes of the phase lag of the free end relative to the beam base. The phase lag is defined as the value of the difference between the phase of the oscillation at the beam free-end and the phase of the excitation at the beam base. The phase of the beam free-end is always behind the phase of the beam base. In the free vibration, the phase lag is about 81°. As the contact deepens, the phase lag decreases to about 18° in the half cycle where there is no interaction force.

**Fig. 10:**
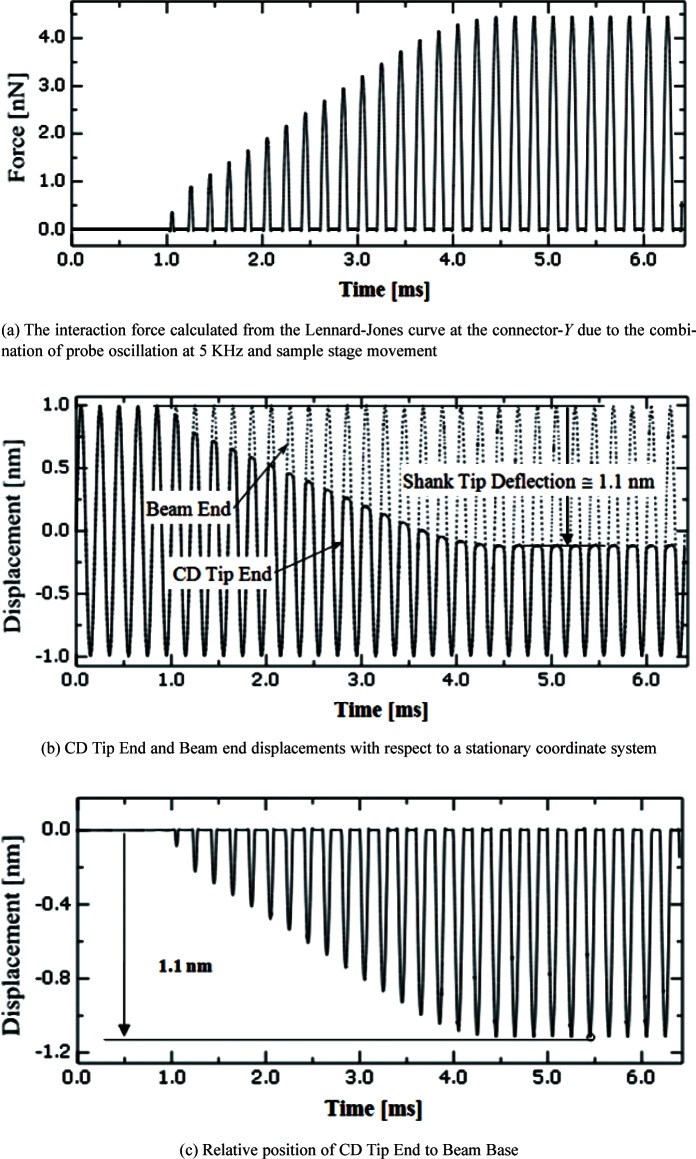
Estimated tip deflection and interaction forces in *Y*.

When an edge of the CD tip end is in contact with a side wall during side wall scanning, the CD tip deflects. Figure 10 shows the interaction force and the CD tip deflection. Figure 10(a) shows that the interaction force increases from zero (no contact) to about 4.4 nN when in full contact. Figure 10(b) shows that the deflection of the tip end relative to the beam end is about 1.1 nm due to the contact force of 4.4 nN in the *Y* direction. During contact with the sample surface, the tip end deflects due to the Lennard-Jones force between the CD tip edge and the surface. Figure 10(c) shows the relative position of the CD probe tip to the beam base. The relative position is obtained by subtracting the beam base position from the CD probe tip position in Fig. 10(b). Since the AFM reading in the *Z* direction is the instant position of the beam base during scanning, this figure shows the tip compliance. The maximum deflection due to tip compliance is 1.1 nm in this example. In this way, the modeling can help metrologists estimate probe compliance and instabilities due to design geometry and materials properties.

**Fig. 11:**
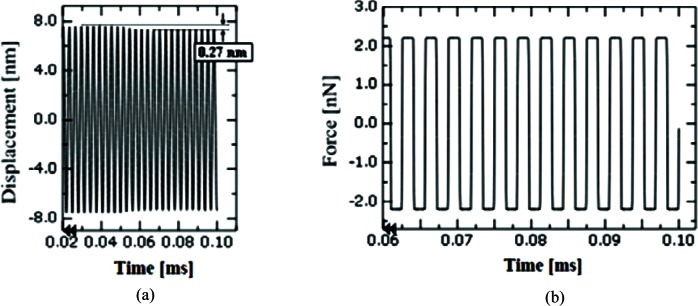
Estimated beam deflection due to a sliding frictional force in probing a side wall.

Figure 11 illustrates the effect of frictional forces in side-wall probing. Assuming the friction coefficient of the CD tip moving on the side wall is 0.5, the frictional force reduces the amplitude of the beam oscillation by about 0.27 nm in the *Z* direction as shown in Fig. 11(a). Due to the beam oscillation, friction force is acting up and down in the *Z* direction as shown in Fig. 11(b). The force magnitude is around 2.2 nN in the *Z* direction, which can be calculated by multiplying the friction coefficient of 0.5 by the maximum contact force of 4.4 nN in the *Y* direction. The assumed friction coefficient of 0.5 is close to the range (0.55 to 4.5) of friction coefficients measured for Si by Chen and Carman [12] for MEMS (Micro Electro Mechanical Systems) scale structures in contact.

**Fig. 12:**
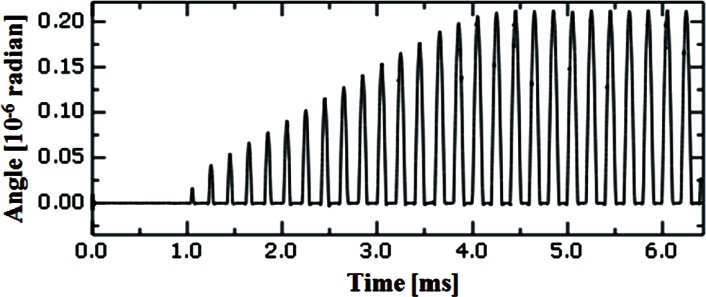
Angular displacement change under the interaction force change similar to Fig. 10 (a).

**Fig. 13:**
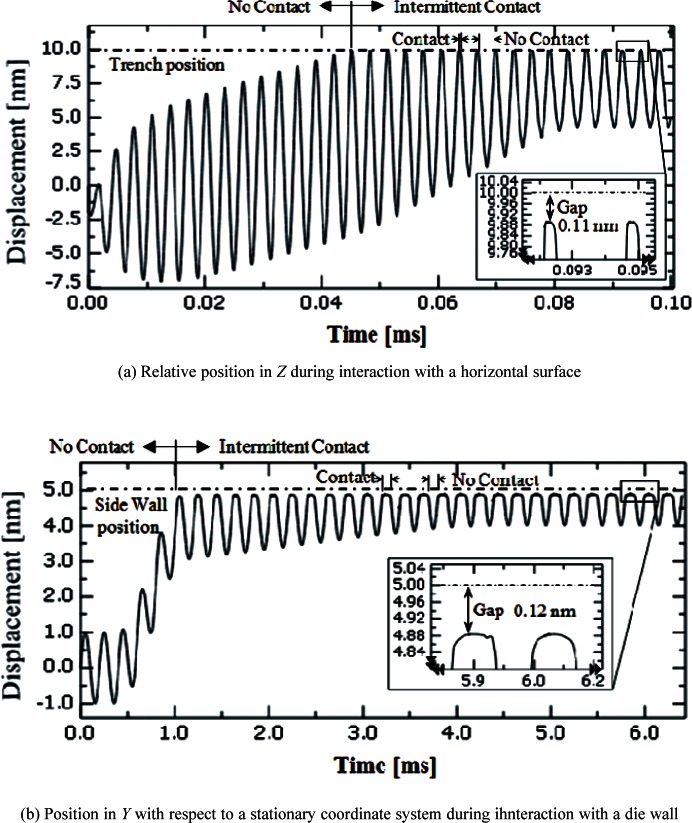
Relative position of the tip end during interaction with a sample.

Even if the friction coefficient is very small, contact can be detected by an angular displacement of the beam end due to twisting caused by the lateral contact force at the tip of the probe. Figure 12 shows the angular displacement about *X* at the beam end. The maximum angular displacement is about 2.12 × 10^−7^ radian, which is generated by the maximum lateral contact force of 4.4 nN at the end of the tip. With the computed CD tip displacement and the defined probe movement (Fig. 4b) in the time domain, the relative distance between the CD tip and the sample surface to be probed can be calculated and plotted. The relative position can be also called “the gap.” Figure 13(a) shows a noncontact region and an intermittent contact region for the horizontal surface case (Fig. 2a). The sample surface is initially 10 nm below the neutral position of the CD tip end. When the tip sample distance is close enough so that an attractive force is applied to the tip, the intermittent contact mode starts. As the sample keeps moving towards the tip, the contacting time increases and repulsive forces are exerted on the tip. At contact, the minimum gap between the CD tip end and the horizontal sample surface is 0.11 nm. Figure 13(b) shows the relative position between the tip end and a side wall in the time domain. The side wall is initially 5 nm apart from the probe tip. The minimum gap between an edge of the CD tip end and the side wall is 0.12 nm.

The measured point on a side wall can be estimated by combining the computed gap between tip and surface, as shown in Fig. 13(b), and the CD tip deflection relative to the beam base, as shown in Fig. 10(c). In this example, the *Y* coordinate of the measured point deviates from the *Y* reading in AFM by 1.22 nm, 0.12 nm gap and 1.1 nm tip deflection (Fig. 10b).

## 4. Conclusions

A new computational model for CD AFM has been developed for the analysis and characterization of static and dynamic behaviors for both side wall and horizontal surface probing. This computational model enables metrologists to analyze and visualize the probe behaviors in intermittent surface contact. Interactions between the CD tip end and sample surface are complex, but interaction forces and relative positions can be computed and plotted to simulate the CD tip-sample surface interactions based on Lennard-Jones forces. To analyze probe compliance, the CD tip deflection is computed and analyzed based on the relative deflection of the probe tip to the beam end. Since it cannot be detected by the laser sensor in CD AFM, the estimated deflection helps predict instabilities in probe behavior due to the probe compliance in scanning. Also, measured points can be estimated using the finite element modeling and analysis with available interaction force models. The Lennard-Jones model used here provides a starting point to which other interaction forces can be added, such as a capillary force.

Some parameters were estimated in the model. Friction coefficients may vary significantly depending on the materials of the CD tip and sample. We chose a friction coefficient of 0.5 using data taken at MEMS (Micro Electro Mechanical Systems) scales [12]. Data for the friction coefficient on the nanometer scale is needed. The damping ratio in the probe used in the model is an estimate. More accurate values should be determined by experiments to further improve the model. It still remains to validate the model in detail by comparison with experiments. However, in previous work, the FEA (Finite Element Analysis) of the cantilever-probe assembly was validated by comparison with analytical calculations [5]. The FEA model there was useful for estimating uncertainty due to probe compliance in research to measure linewidth using a conventional, top-down AFM probe with a nanotube tip [13]. Furthermore, using reasonable assumptions here for the cantilever parameters and interaction forces has led to insightful results for calculated deflection amplitudes, deflection waveforms vs. time, and resonant frequencies, in line with the expected properties of these systems. Possible future work includes studying the interaction during transitions from scanning a horizontal surface to a vertical surface, and from a vertical surface to a horizontal surface. Also, including more surface area on the side of the CD tip in the Lennard-Jones interaction and capillary forces will provide better estimation of the Lennard-Jones forces.

## Figures and Tables

**Table 1:** The first five natural frequencies of the CD-AFM probe

Mode	Frequency [kHz]
First mode (flexural)	328.0
Second mode (flexural)	2071.0
Third mode (lateral)	2357.7
Fourth mode (torsional)	2836.4
Fifth mode (flexural)	5819.9

## Footnotes

2 No approval or endorsement of any commercial products by the National Institute of Standards and Technology (NIST) is intended or implied. Certain commercial software systems are identified in this paper to facilitate understanding. Such identification does not imply that these systems are necessarily the best available for the purpose.
